# Sperm function, mitochondrial activity and in vivo fertility are associated to their mitochondrial DNA content in pigs

**DOI:** 10.1186/s40104-023-00988-0

**Published:** 2024-02-01

**Authors:** Marc Llavanera, Yentel Mateo-Otero, Estel Viñolas-Vergés, Sergi Bonet, Marc Yeste

**Affiliations:** 1 Unit of Cell Biology, Department of Biology, Faculty of Sciences, University of Girona, Girona, ES-17003 Spain; 2 Biotechnology of Animal and Human Reproduction (TechnoSperm), Institute of Food and Agricultural Technology, University of Girona, Girona, ES-17003 Spain; 3 Catalan Institution for Research and Advanced Studies (ICREA), Barcelona, ES-08010 Spain

**Keywords:** Fertility, Mitochondria, Motility, mtDNA copy number, qPCR, Sperm

## Abstract

**Background:**

Despite their low abundance in sperm, mitochondria have diverse functions in this cell type, including energy production, signalling and calcium regulation. In humans, sperm mitochondrial DNA content (mtDNAc) has been reported to be negatively linked to sperm function and fertility. Yet, the association between mtDNAc and sperm function in livestock remains unexplored. For this reason, this study aimed to shed some light on the link between mtDNAc and sperm function and fertilising potential in pigs. A qPCR method for mtDNAc quantification was optimised for pig sperm, and the association of this parameter with sperm motility, kinematics, mitochondrial activity, and fertility was subsequently interrogated.

**Results:**

First, the qPCR method was found to be sensitive and efficient for mtDNAc quantification in pig sperm. By using this technique, mtDNAc was observed to be associated to sperm motility, mitochondrial activity and in vivo, but not in vitro, fertility outcomes. Specifically, sperm with low mtDNAc were seen to exhibit greater motility but decreased mitochondrial activity and intracellular reactive oxygen species. Interestingly, samples with lower mtDNAc showed higher conception and farrowing rates, but similar in vitro fertilisation rates and embryo development, when compared to those with greater mtDNAc.

**Conclusions:**

These findings enrich our comprehension of the association of mtDNAc with sperm biology, and lay the foundation for future research into employing this parameter as a molecular predictor for sperm function and fertility in livestock.

**Supplementary Information:**

The online version contains supplementary material available at 10.1186/s40104-023-00988-0.

## Introduction

Mitochondria are organelles known to be responsible for a wide range of cellular functions, such as energy production, signalling pathway regulation and calcium homeostasis [1]. In spite of this, their role in sperm is complex and still partially unknown. Indeed, it is worth noting that sperm have a highly differentiated structure consisting of a unique, specialised sheath in the midpiece that gathers all mitochondria; suggesting that this organelle has a crucial function during sperm life [2, 3]. Mitochondria are characterised by containing their own genome structured in a circular, double-stranded DNA molecule that encodes 13 protein subunits of the electron transport chain (ETC), 22 tRNAs and 2 rRNAs [4]. Replication of mitochondrial DNA (mtDNA) is regulated by mitochondrial transcription factor A (TFAM) and mitochondrial-specific DNA polymerase gamma (POLG [5, 6]). Both TFAM and POLG are known to be downregulated during spermatogenesis, thus coinciding with the reduction of mtDNA content (mtDNAc) after this process [7–9]. Moreover, during spermiogenesis, most of the cytoplasm of spermatids is eliminated through the formation of residual bodies [10, 11]. After these two processes, therefore, the vast majority of mitochondria are lost, with the consequent depletion of mtDNA copies. In effect, a small number of these organelles is confined to the sperm midpiece [11], which explains why the average number of mtDNA copies in human sperm ranges between 1 and 1,000 [1, 12]. Interestingly, Wai et al. [13] generated germline-specific *Tfam* heterozygous mouse males to reduce mtDNAc in sperm. The results showed that sperm can tolerate a threefold reduction in mtDNAc without any impairment to their function. Nevertheless, the unprotected nature of the mitochondrial genome makes it 10–100 times more susceptible to oxidative damage than nuclear DNA [11, 14], since mtDNA is devoid of protective histones and close to the ROS generated at the ETC as a by-product of oxidative phosphorylation [15].

Previous studies conducted in human sperm evaluated the relationship of mtDNAc with quality and fertility parameters. Sperm mtDNAc was reported to be negatively associated with seminal quality in several studies [1, 16–18]. A recent systematic review and meta-analysis evidenced a higher amount of mtDNA in sperm of men with abnormal semen analysis compared to those showing a normal seminogram [19]. In accordance with these results, the higher the mtDNAc in sperm the poorer the quality [20]. While these findings suggest that quantifying mtDNAc in sperm may predict the male reproductive status in humans, few studies on this subject have been conducted in farm animals. Remarkably, the previous research performed in horses and pigs also concurs that mtDNAc in sperm is negatively associated to sperm quality [21, 22]. Yet, whether mtDNAc is related to sperm function, mitochondrial activity, and in vitro and in vivo fertility has not been investigated in pig sperm.

In the light of the above, the present study aimed to elucidate if mtDNAc in sperm is linked to their motility, kinematics, mitochondrial activity, and fertility in pigs.

## Materials and methods

### Reagents, samples and experimental design

All chemicals were purchased from Sigma-Aldrich Chemical (St Louis, MO, USA) unless otherwise indicated. All fluorochromes (SYBR^®^ 14, propidium iodide [PI], 1,1’,3,3’-tetraethyl-5,5’,6,6’-tetrachloroimidacarbocyanine iodide [JC-1], dihydroethidium [HE] and Yo-Pro-1) were purchased from Thermo Fisher Scientific (Waltham, MA, USA).

Seminal doses intended to artificial insemination (AI) were produced following the current European Union legislation for animal health, welfare and husbandry. Seminal doses were procured from an AI-centre (Grup GePork, Les Masies de Roda, Spain). To prepare these doses, 22 ejaculates from separate healthy and sexually mature boars (*n* = 22; each ejaculate came from a different animal) were collected using the gloved hand method. Subsequently, ejaculates were diluted in a commercial extender (Duragen; Magapor, Ejea de los Caballeros, Spain) to achieve a final concentration of 33 × 10^6^ sperm/mL. Samples were then carefully maintained at 17 ºC. One seminal dose per male was transported to the laboratory within 4 h post-collection.

Upon arrival at the laboratory, seminal doses were split into 3 aliquots. The first aliquot was used to evaluate sperm function through a computer-assisted sperm analysis (CASA) system (sperm motility and kinematics) and a flow cytometer (mitochondrial membrane potential [MMP] and intracellular superoxide [•O_2_^−^] levels. The second aliquot was utilised to quantify mtDNAc by quantitative PCR (qPCR) targeting both nuclear and mitochondrial-encoded genes. The third aliquot was used to perform IVF experiments in order to evaluate the in vitro fertilising ability of each sperm sample. The remaining seminal doses produced at the AI-center from 13 of the 22 boars were employed to conduct AI for commercial purposes, and data were collected to evaluate in vivo fertility.

### Sperm motility assessment

Sperm samples were kept at 38 °C for 10 min and loaded onto pre-warmed 20-µm Leja chamber slides (Leja Products BV; Nieuw-Vennep, The Netherlands). Motility parameters were recorded using a CASA system consisting of a negative phase-contrast field microscope (Olympus BX41 with 10 × 0.30 PLAN objective; Olympus, Tokyo, Japan) and a computer equipped with ISAS software (Integrated Sperm Analysis System V1.0; Proiser SL, Valencia, Spain). A total of 500 sperm per replicate were analysed. Sperm with an average path velocity (VAP; µm/s) higher than 10 μm/s were considered motile, and those with an index of straightness (STR; straight-line velocity (VSL)/VAP; %) higher than 45% were classified as progressively motile. Proportions of total (TMOT; %) and progressively (PMOT; %) motile sperm were used to evaluate their motility. Sperm kinematics was assessed on the basis of average path velocity (VAP; µm/s), curvilinear velocity (VCL; µm/s), straight-line velocity (VSL; µm/s) and linearity (LIN; VSL/VCL; %).

### Evaluation of sperm viability, mitochondrial activity and superoxide levels with flow cytometry

Flow cytometry was used to analyse sperm viability, MMP and intracellular •O_2_^−^ levels. Sperm samples were diluted to 2 × 10^6^ sperm/mL and stained following the corresponding protocol before analysis with a CytoFLEX cytometer (Beckman Coulter; Fullerton, CA, USA). The sperm population was gated using forward (FSC) and side scatter (SSC), and a minimum of 10,000 sperm events were examined. All fluorochromes were excited using a 488 nm laser. Fluorescence from SYBR^®^ 14, Yo-Pro-1 and JC-1 monomers (JC-1_mon_) was acquired by the FITC channel (525/40), fluorescence from dihydroethidium (HE) and JC-1 aggregates (JC-1_agg_) was detected through the PE channel (585/42), and fluorescence from propidium iodide (PI) was collected through the PC5.5 channel (690/50). The gain and flow rate remained constant throughout the experiment.

Sperm viability was determined following the protocol of Garner and Johnson [23]. Sperm were incubated with 32 nmol/L SYBR^®^ 14 and 7.6 µmol/L PI in the dark, at 38 °C for 10 min. The proportion of SYBR^®^ 14-positive and PI-negative (SYBR^®^ 14^+^/PI^−^) cells, after subtracting the proportion of debris particles, was used to evaluate sperm viability.

The MMP in sperm was evaluated following the protocol described by Llavanera et al. [24]. Sperm were incubated with 750 nmol/L JC-1 in the dark, at 38 °C for 30 min. A high membrane potential within the mitochondria leads to the formation of JC-1_agg_, whereas JC-1_mon_ are present in mitochondria with low membrane potential. The ratio between the mean fluorescence intensities of JC-1_agg_ and JC-1_mon_ was used to evaluate MMP in sperm.

Intracellular •O_2_^−^ levels in sperm were determined following the procedure described by Guthrie and Welch [25], with minor modifications. Sperm were incubated with 5 µmol/L HE and 31.3 nmol/L Yo-Pro-1 in the dark, at 38 °C for 20 min. The mean fluorescence intensity of ethidium (E^+^) was used to evaluate •O_2_^−^ levels in sperm.

### Quantification of sperm mitochondrial DNA (mtDNA) content

The content of mtDNA was evaluated by quantitative PCR (qPCR) through the relative quantification of genes encoded in nuclear (BCL2 Associated X; *BAX*) and mitochondrial (NADH dehydrogenase subunit 1; *ND1*) genomes [16]. A total of 100 × 10^6^ sperm per sample were centrifuged at 2,000 × *g* and room temperature for 5 min, and resuspended in PBS. Isolation of total sperm DNA was carried out with the DNeasy kit (Qiagen, Hilden, Germany) following the manufacturer’s recommendations. The lysis buffer was supplemented with 50 mmol/L dithiothreitol (DTT) with the aim to break the disulphide bridges of the mitochondrial capsule [16, 26]. DNA concentration and quality were subsequently quantified using an Epoch Microplate Spectrophotometer (Vermont, USA). The A_260__/__280_ quotient was ~ 1.8 in all samples. Following this, *BAX* and *ND1* genes were analysed by qPCR using the SYBR^®^ Select Master Mix (Applied Biosystems, CA, USA), as recommended by the manufacturer. Forward and reverse primers employed in this study were designed with Primer Blast [27] and are shown in Table 1. Specificity of amplifications was evaluated by melting curves and 2% agarose gel electrophoresis (Additional file 1), whereas amplification efficiency of qPCR was determined by primer efficiency analysis (Table 1; [28]). The impact of density-gradient centrifugation (DGC) of semen on the quantification of mtDNAc in sperm was also evaluated (Additional file 2). The qPCR assay was performed in a total volume of 20 µL, containing 10 µL of SYBR^®^ Select Master Mix, 375 nmol/L of forward and reverse primers and 15 ng of DNA. Samples were run in triplicate and a no template control (NTC) was included in every reaction plate. The mean standard deviation (SD) of technical replicates was ± 0.11 (from 0.01 to 0.51). DNA amplification was developed in an Applied Biosystems 7500 Real-Time PCR device (Thermo Fisher), and thermo-cycling conditions were as follows: incubation at 50 °C for 2 min; incubation at 95 °C for 2 min; 40 cycles of 15 min at 95 °C and 1 min at 60 °C; and a melt curve analysis consisting of 15 s at 95 °C, 1 min at 60 °C, 30 s at 95 °C and 15 s at 60 °C. Relative mtDNA content was calculated using the resulting qPCR data (mean of the cycle threshold (C_T_) from triplicates) applying the formula described by Rooney et al. [29]:

1 $$\triangle{\mathrm C}_{\mathrm T}=\left(\mathrm{nucDNA}\;{\mathrm C}_{\mathrm T}-\mathrm{mtDNA}\;{\mathrm C}_{\mathrm T}\right)$$

2 $$\mathrm{Relative}\;\mathrm{mtDNA}\;\mathrm{content}=2\;\mathrm \times\;2^{\triangle{\mathrm C}_{\mathrm T}}$$

**Table 1:** Primer details for quantifying sperm mitochondrial DNA content (mtDNAc) by qPCR

Genome	Target	Primer sequence (5´→3´)	Tm, °C	Amplicon size, bp	Primer efficiency
Nuclear	*BAX*	FW: GGACTTCCTTCGAGATCGGC	60	161	94.81
		RV: GAGCACATCTGGTGACCCAA			
Mitochondrial	*ND1*	FW: CCATGTTCATTATTGCACCAATCC	60	190	109.88
		RV: GCCCCGATGAGTGCGTATTT			

Sequence, melting temperature (Tm), amplicon size and efficiency of the primers used to amplify the nuclear (BCL2 Associated X; *BAX*) and mitochondrial (NADH dehydrogenase subunit 1; *ND1*) genes. The number of copies of these genes were subsequently utilised to quantify mitochondrial DNA content (mtDNAc) in pig sperm. *FW* Forward; *RV* Reverse

### In vitro maturation (IVM), fertilisation (IVF) and embryo culture (IVC)

Ovaries of prepubertal gilts were obtained from a local abattoir (Frigoríficos Costa Brava, S.A., Riudellots de la Selva, Spain), kept in a physiological saline solution with 70 µg/mL kanamycin at 38 °C, and transported to the laboratory within 2 h of collection. Upon arrival, cumulus-oocyte complexes (COCs) were retrieved from ovarian follicles, washed in Dulbecco’s PBS (Gibco, Thermo Fisher), and subjected to IVM. Clusters of 50–60 immature COCs were placed in a multidish (Nunc, Roskilde, Denmark), and incubated in in vitro maturation (IVM) medium, consisting of TCM-199 (Gibco) supplemented with 0.57 mmol/L cysteine, 0.1% (w:v) polyvinyl alcohol, 10 ng/mL human epidermal growth factor, 75 µg/mL penicillin-G potassium, 50 µg/mL streptomycin sulphate, 10 IU/mL equine chorionic gonadotropin (eCG; Folligon; Intervet International B.V.; Boxmeer, The Netherlands) and 10 IU/mL human chorionic gonadotropin (hCG; Veterin Corion; Divasa Farmavic S.A.; Gurb, Barcelona, Spain). After 22 h of incubation at 38.5 °C in 5% CO_2_ and 95% relative humidity, COCs were moved to a hormone-free IVM medium (without eCG or hCG) and incubated further for 20–22 h. Then, in vitro matured COCs were treated with 0.05% hyaluronidase in Dulbecco’s PBS (Gibco, Thermo Fisher) and mechanically pipetted for decumulation. Subsequently, oocytes were placed into 50-µL drops of in vitro fertilisation (IVF) medium (modified from Tris-buffered medium [30]) supplemented with 1 mmol/L caffeine. Next, IVF was carried out by adding 1,000 sperm per oocyte, and gametes were co-incubated at 38.5 °C in 5% CO_2_ and 95% relative humidity for 5 h. Two technical replicates of 50 oocytes per semen sample were inseminated. The presumptive zygotes were then washed and transferred to in vitro culture (IVC) medium consisting of NCSU23 medium [31] supplemented with 0.4% BSA, 0.3 mmol/L pyruvate and 4.5 mmol/L lactate. After 2 d of incubation, fertilisation rates were calculated by counting cleaved embryos. Thereafter, all putative embryos were transferred into NCSU23 medium supplemented with 0.4% BSA and 5.5 mmol/L glucose and incubated further for 4 d. The resulting embryos were classified following the criteria established by Balaban and Gardner [32]. The percentage of cleaved embryos at d 2 (fertilisation rate; %) and the percentage of embryos at d 6 (total number of embryos; %) were calculated to determine in vitro fertilisation ability and embryo development.

### Artificial insemination

In vivo reproductive performance of 13 boars was evaluated by carrying out AI on a total of 1,149 multiparous sows in a breeding farm (Granja La Lluçanesa; Prats de Lluçanes, Barcelona, Spain). Sows were housed in environmentally-regulated facilities, and supplied with an adjusted diet and water ad libitum. Oestrus in females was evaluated on the basis of vulvar reddening and enlargement, as well as their responsiveness to a male teaser. Oestrus manifestation was validated at four and 5 d post-weaning, applying a gentle pressure on the sow’s dorsal region to ascertain the occurrence of the standing reflex. A responsive reaction to dorsal pressure defined the initiation of oestrus. AI was conducted through post-cervical insemination using a Magaplus S catheter (Magapor; Zaragoza, Spain), and an average of 88 AIs per boar was carried out. In vivo fertility parameters including conception rate (evaluated after 30 d through ultrasonography; Echoscan T-100, Import-Vet, S.A.; Barcelona, Spain) and farrowing rate (the proportion of inseminated sows that farrowed) were recorded.

### Statistical analysis

Data were plotted through GraphPad Prism 8.0 Software (GraphPad, San Diego, USA) and analysed through SPSS v. 27.0 (IBM Corp.; Armonk, NY, USA). Pearson correlations between mtDNAc and sperm function variables were calculated. Moreover, a hierarchical cluster analysis using Euclidean distances and the Bayesian information criterion (BIC) led to the classification of sperm samples into two groups (low or high mtDNAc). In vitro fertility parameters (fertilisation rate and embryo development) and in vivo fertility outcomes (farrowing and conception rates) were logit-transformed [logit = ln(*x*/1 - *x*)] and the resulting log-odds were used for further comparisons between groups. Following this, normal distribution was determined through the Shapiro–Wilk test, whereas homogeneity of variances was examined through the Levene test. In vitro fertility parameters (fertilisation rate and embryo development) were compared between groups with a Student’s *t*-test. In vivo fertility outcomes (farrowing and conception rates) were weighted considering the number of inseminations involving each male, and compared between groups using the Mann–Whitney U test as data did not match parametric assumptions. For all tests, the level of significance was set as *P* ≤ 0.05.

## Results

### Quantification of *ND1 *and *BAX *by qPCR can be used to assess mtDNAc in pig sperm

First, a method for the relative quantification of mtDNAc was optimised for pig sperm. As shown in Table 1, primer efficiency for the amplification of both *ND1* and *BAX* varied between 90 and 110. Moreover, considering the presence of a single sharp peak in melting curve analysis and the presence of single-band patterns of ~ 190 and ~ 160 bp in agarose gels, respectively, the primers designed for both genes were considered as highly specific (Additional file 1).

### Sperm with higher mtDNAc show impaired motility and kinematic parameters

**Fig. 1:**
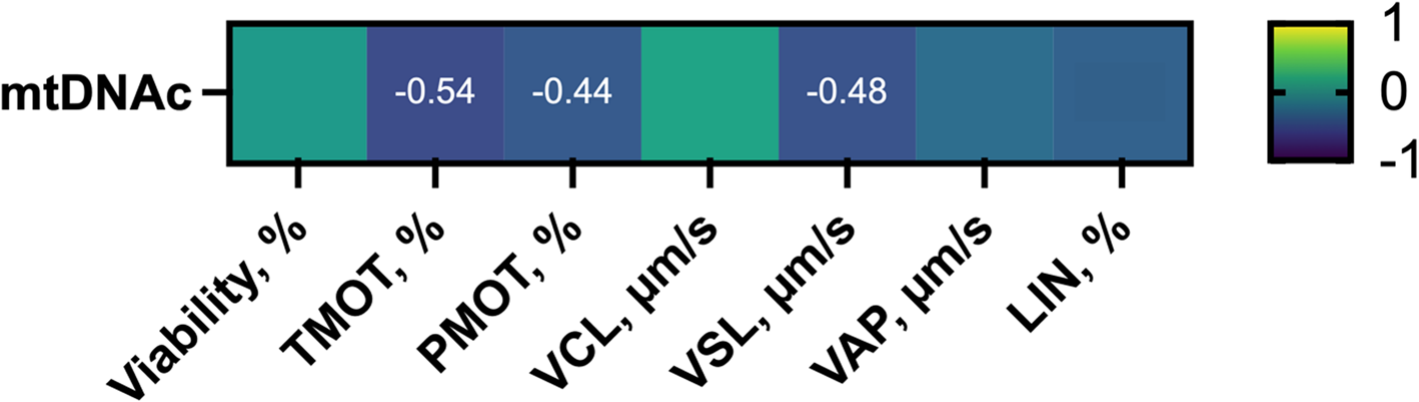
Heat map of Pearson correlation coefficients (*r*) between mitochondrial DNA content (mtDNAc) and sperm function parameters (sperm viability, motility, and kinematic parameters). *n* = 22. The presence of the *r* value in a cell indicates statistically significant correlations (*P* < 0.05)

The next aim of the present study was to explore the relationship of mtDNAc in sperm with sperm motility, kinematics and viability (Fig. 1). Pearson correlation showed a negative correlation between mtDNAc in sperm and their TMOT (*r* = −0.54; *P* < 0.05), PMOT (*r* = −0.44; *P* < 0.05) and VSL (*r* = −0.48; *P* < 0.05). In contrast, mtDNAc was found not to be associated to VCL, VAP, LIN or sperm viability (*P* > 0.05).

### Sperm mitochondrial activity is correlated to mtDNAc

**Fig. 2:**
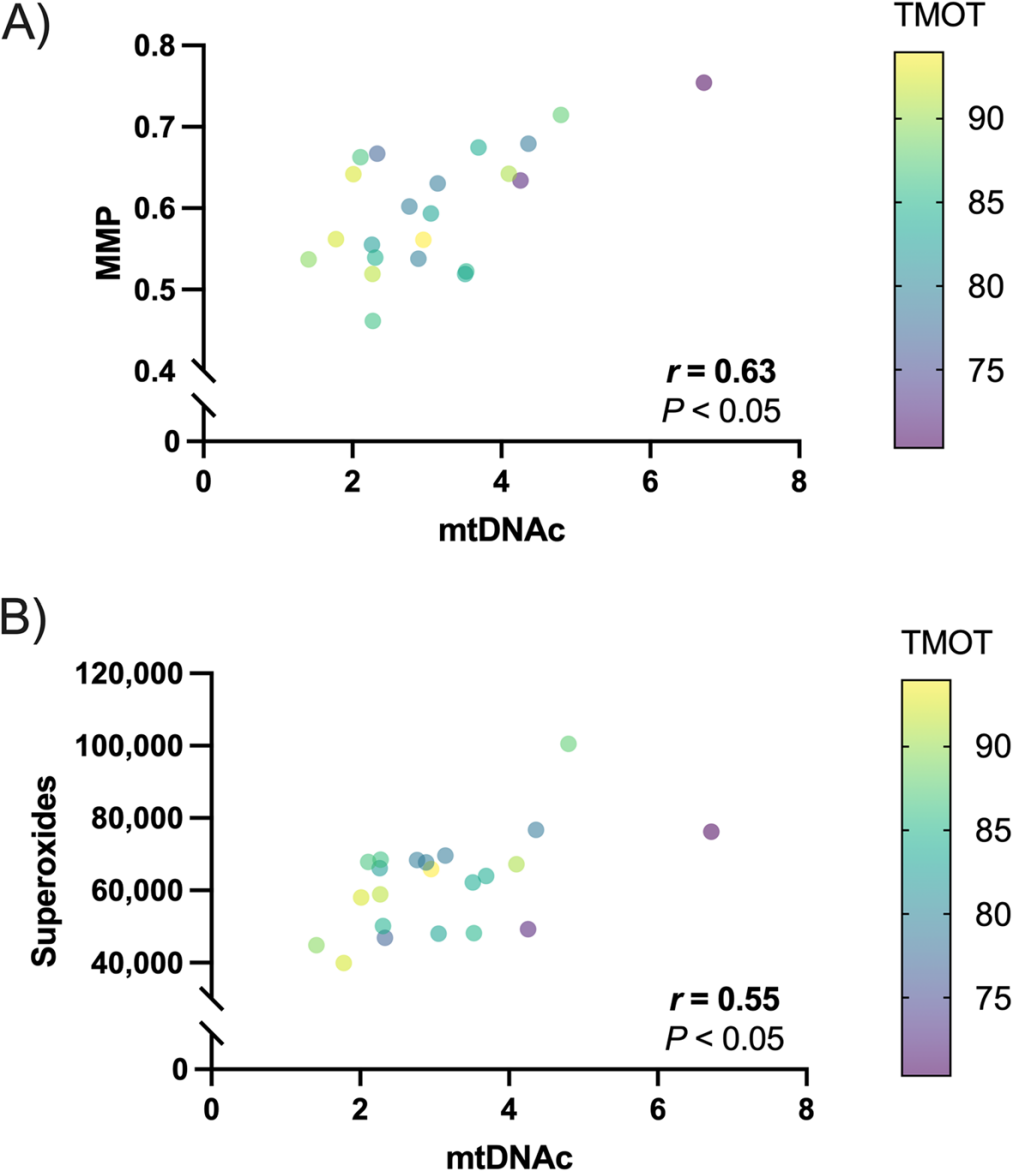
Correlation matrix of (**A**) mitochondrial membrane potential (MMP) and (**B**) intracellular superoxide levels (superoxides) in sperm with their mitochondrial DNA content (mtDNAc) and total motility (TMOT). Pearson correlation coefficients (*r*) and *P*-values correspond to the correlation between each mitochondrial-associated parameter (i.e., MMP and superoxide levels) and mtDNAc in sperm. *n* = 22

Whether mtDNAc in sperm is related to mitochondrial function was investigated by evaluating MMP and intracellular levels of •O_2_^−^ (Fig. 2). The correlation matrix evidenced a positive correlation of mtDNAc in sperm with MMP (*r* = 0.48; *P* < 0.05) and •O_2_^−^ levels (*r* = 0.47; *P* < 0.05). In both cases, interestingly, TMOT of sperm was significantly and negatively correlated with their mtDNAc (*r* = −0.54; *P* < 0.05). Altogether, the results revealed that not only did sperm with higher mtDNAc have greater mitochondrial activity and •O_2_^−^ levels but also lower motility.

### mtDNAc in sperm is associated with in vivo, but not in vitro fertility outcomes

**Fig. 3:**
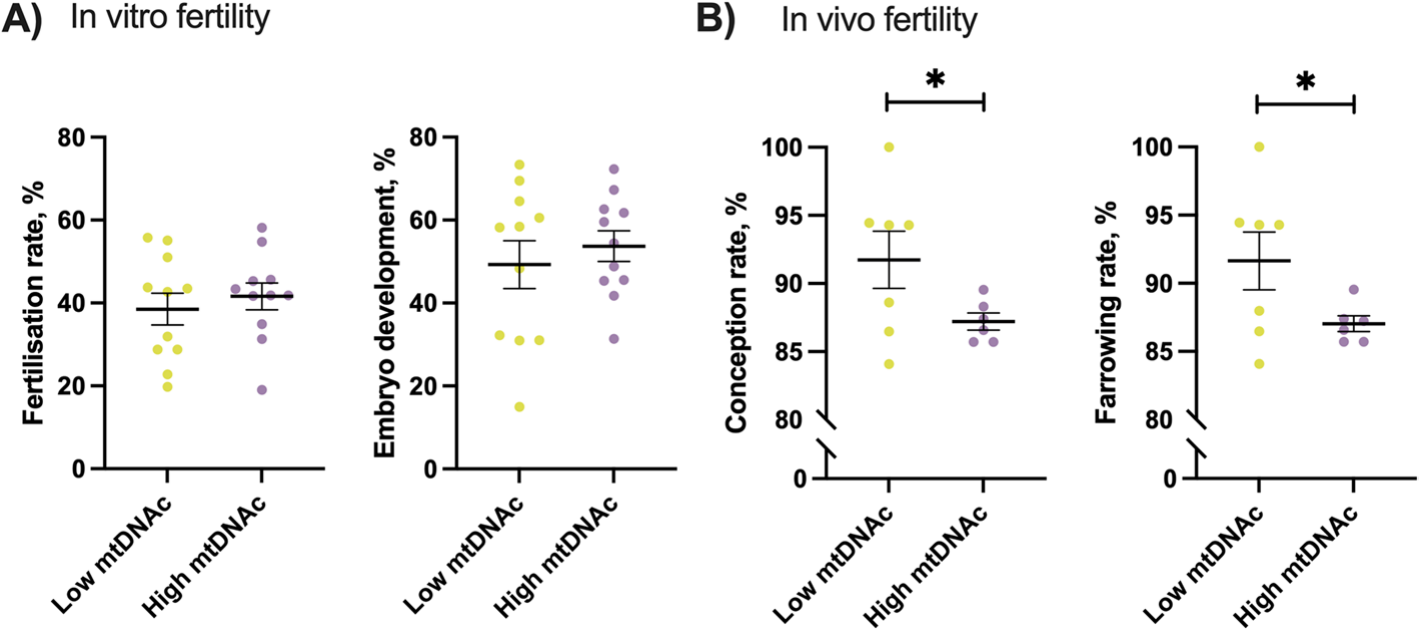
Mean and standard error of the mean (SEM) of (**A**) in vitro fertility parameters (fertilisation rate and embryo development) and (**B**) in vivo fertility outcomes (conception rate and farrowing rate) between sperm samples classified as having low or high mitochondrial DNA content (mtDNAc). *n* = 22 for in vitro fertility outcomes and *n* = 13 for in vivo fertility outcomes. ^*^P < 0.05

Considering the correlation between mtDNAc and sperm function variables, the putative association between this parameter and the fertilising ability of pig sperm was then investigated. As shown in Fig. 3, sperm samples with low and high mtDNAc exhibited similar oocyte fertilisation rates and embryo development (*P* > 0.05). Interestingly, however, conception and farrowing rates were greater in sperm samples exhibiting low mtDNAc than in those with high mtDNAc (*P* < 0.05).

## Discussion

As evidenced by previous studies in various species, including humans and horses, determining mtDNAc in sperm has garnered significant attention because of its association with sperm quality and function [1, 16–18, 21]. Yet, little is known about the relationship between mtDNAc and both sperm function and fertilising ability in pigs. In a previous study conducted in swine [22], intra-ejaculate cohorts of motile and sub-motile sperm were assessed after density gradient centrifugation to investigate the correlation between mtDNAc, motility, and mitochondrial activity. The authors reached the conclusion that motile sperm exhibit higher mitochondrial activity and ROS production, while concomitantly showing lower mtDNAc when compared to sub-motile sperm within the same ejaculate. Nonetheless, this approach did not evaluate the relationship between mtDNAc and fertility, and overlooked potential inter-ejaculate differences. Consequently, the present study aimed to complement these previous findings by examining the association between mtDNAc, sperm function, and fertility potential.

The quantification of the mitochondrial- and nuclear-encoded genes evaluated here was previously employed for the quantification of mtDNAc in human sperm [16]. To the best of our knowledge, the current work is the first to optimise a qPCR analysis for the relative quantification of mtDNAc in pigs, using *ND1* and *BAX* genes. Herein, the sensitivity of the designed primers, as well as their efficiency to amplify the target DNA, was observed to be suitable for the quantification of mtDNAc in this species. One can thus consider that this is a sensitive, efficient, and rapid methodology to evaluate the mtDNAc in pig sperm. On the other hand, our relative quantification of mtDNAc in pig sperm suggests a very low number of mtDNA copies per cell in this species. Although the average number of mtDNA copies is known to range between 1 and 1,000 copies per cell in human sperm [1, 12], this number seems to be lower in pig sperm. Significant differences in terms of sperm mtDNAc have been reported across species since, for instance, mtDNA is known to be completely depleted during sperm elongation in invertebrates [1, 33, 34]. Moreover, worthy of notice is that our results are based on a relative quantification of mtDNAc, so that additional studies using an absolute quantification method are needed to compare mtDNA copy number in sperm between mammalian species.

Previous studies associated higher mtDNAc in sperm with diminished motility in humans [1, 19] and horses [21]. Similarly, the current study in pigs demonstrated a negative correlation between sperm mtDNAc and both their motility and velocity. These findings substantiate an evident relationship between sperm mtDNAc and their motile capacity, which appears to be conserved across mammalian species. Yet, the mechanisms underlying the association between mtDNAc and sperm motility are still uncovered. Considering the statistically significant correlation observed between mtDNAc and sperm motility in mammals, the current study, in a similar fashion to previous research [1, 19, 21], explored the association between sperm motility and mitochondrial activity. As previously mentioned, in a preceding study conducted in pigs, distinct intra-ejaculate cohorts of motile and sub-motile sperm were selected; this was followed by an assessment of their mtDNAc, motility and mitochondrial activity [22]. That study showed that motile sperm exhibited lower content of mtDNA and higher mitochondrial activity and ROS production compared to sub-motile sperm. In the present work, a comparative analysis of mtDNAc and sperm mitochondrial activity was extended to an inter-ejaculate context. Similarly, the findings reported herein pointed out that, in spite of the negative association of mtDNAc in sperm with motility and velocity, a positive correlation was obvious between mtDNAc and both mitochondrial activity and intracellular •O_2_^−^ levels. Interestingly, the previous and the current study converge on the conclusion that, despite observing lower mtDNAc in highly motile sperm, these cells show higher mitochondrial activity and ROS production. This suggests a link between mtDNAc, sperm motility, and mitochondrial function. Up to the present time, two main hypotheses have been proposed to explain these findings. Firstly, increased mtDNAc in impaired sperm samples may be attributed to defective spermiogenesis leading to a deficient regulation of mtDNA replication [20, 35]. The second hypothesis suggests that, in sperm with impaired physiology and energy production, a compensatory effect could occur, with the overexpression of *TFAM* or *POLG* underlying the higher mtDNAc [36]. Either way, it is evident that the quantification of sperm mtDNAc represents an indirect assessment of the physiological state of these cells, thus making this parameter an ideal molecular marker. It is, nevertheless, worth indicating that the low number of mtDNA copies per cell in pig sperm may limit the association of this parameter with sperm physiology.

Finally, the relationship between mtDNAc and fertility outcomes was evaluated through in vitro fertilisation and artificial insemination. As far as we know, this is the first study evaluating the relationship between sperm mtDNAc and both in vitro and in vivo fertility outcomes in pigs. Interestingly, and notwithstanding the reduced sperm motility, sperm displaying high mtDNAc showed similar outcomes after in vitro fertilisation when compared to those exhibiting low mtDNAc. Coincident results were reported in humans, as mtDNAc present in sperm was not observed to be associated to clinical outcomes after either in vitro fertilisation or intracytoplasmic sperm injection (ICSI; [17]). Remarkably, in humans, in a similar fashion to that observed in the current research in pigs, sperm with high mtDNAc were less motile than those with low mtDNAc but led to similar in vitro fertilisation outcomes. This apparent biological inconsistency can be ascribed to the intrinsic in vitro characteristics of assisted reproductive technologies, as these methodologies circumvent various selection mechanisms inherent to in vivo fertilisation, including sperm competition and the impact of the female reproductive tract milieu. Furthermore, although IVF was designed to mimic in vivo conditions, this procedure entails a series of limitations. The main weakness of IVF, especially in pigs, is a high degree of polyspermy [37]. Consequently, one should not exclude that potential differences between sperm of separate mtDNAc groups in terms of in vitro fertility outcomes could have been masked by other factors inherent to the technique, such as the occurrence of polyspermy.

The link between mtDNAc and in vivo fertility in pigs was also investigated. Previous research in humans reported higher mtDNAc in sperm samples of infertile male when compared to fertile donors [16, 38]. In a similar way, the present study revealed poorer farrowing and conception rates for samples of higher mtDNAc. Yet, a high heterogeneity in terms of in vivo fertility outcomes in the low, but not high, mtDNAc group was noticed. The observed diminished in vivo fertility in samples exhibiting greater mtDNAc could be linked to the decline in sperm motility brought about by heightened mitochondrial activity and the subsequent generation of ROS. In agreement with this hypothesis, previous works reported a negative association between mtDNAc and nuclear DNA integrity in human sperm [18, 39], which could be attributed to increased mitochondrial activity and ROS production. In spite of this, additional investigations involving larger sample sets and a sensitive assessment of sperm DNA integrity should be carried out before reaching robust conclusions about the relationship between mtDNAc and in vivo fertility.

## Conclusions

In conclusion, this study investigated the relationship between sperm mtDNAc and their function and reproductive success in pigs. By optimizing a qPCR method for mtDNA quantification, a sensitive and efficient tool for evaluating mtDNAc in pig sperm was established. The present work also revealed that pig sperm with high mtDNAc exhibit greater mitochondrial activity and intracellular ROS levels, but reduced motility. Notably, this study extended its analysis to fertility outcomes, indicating that mtDNAc in sperm is linked to in vivo but not to in vitro fertilisation outcomes, potentially due to compromised motility stemming from heightened mitochondrial activity and ROS production. The data presented here contribute to the understanding of the link between mtDNAc and sperm biology, and provide a foundation for future investigations into this parameter as a novel molecular marker to evaluate sperm function and in vivo fertility in pigs.

### Supplementary Information

**Additional file 1.** Melt curve analysis and 2% agarose gel electrophoresis of the PCR product resulting from the amplification of the NADH dehydrogenase subunit 1 (*ND1*) and the BCL2 Associated X (*BAX*) genes.

**Additional file 2.** Evaluation of the impact of density-gradient centrifugation (DGC) of semen on the mitochondrial DNA content (mtDNAc) in pigs.

## Data Availability

The datasets used and/or analysed during the current study are available from the corresponding author on reasonable request.

## Abbreviations

•O_2_^−^: Superoxides
BAX: BCL2 associated X
BIC: Bayesian information criterion
CASA: Computer-assisted sperm analysis
COCs: Cumulus-oocyte complexes
CT: Cycle threshold
DGC: Density gradient centrifugation
DNA: Deoxyribonucleic acid
DTT: Dithiothreitol
eCG: Equine chorionic gonadotropin
ETC: Electron transport chain
FW: Forward
hCG: Human chorionic gonadotropin
HE: Dihydroethidium
ICSI: Intracytoplasmic sperm injection
IVC: In vitro culture
IVF: In vitro fertilisation
IVM: In vitro maturation
JC-1: 1,1',3,3'-Tetraethyl-5,5',6,6'-tetrachloroimidacarbocyanine iodide
LIN: Linearity
MMP: Mitochondrial membrane potential
mtDNA: Mitochondrial DNA
mtDNAc: Mitochondrial DNA content
ND1: NADH dehydrogenase subunit 1
NTC: No template control
PBS: Phosphate buffered saline
PI: Propidium iodide
PMOT: Progressive motility
POLG: Mitochondrial-specific DNA polymerase gamma
qPCR: Quantitative polymerase chain reaction
ROS: Reactive oxygen species
rRNA: Ribosomal ribonucleic acid
RV: Reverse
STR: Index of straightness
TFAM: Mitochondrial transcription factor A
TMOT: Total motility
tRNA: Transfer ribonucleic acid
VAP: Average path velocity
VCL: Curvilinear velocity
VSL: Straight-line velocity
