# Supplementary figures and images for: Sperm function, mitochondrial activity and in vivo fertility are associated to their mitochondrial DNA content in pigs
Source: J Anim Sci Biotechnol. 2024 Feb 1;15:10. doi: 10.1186/s40104-023-00988-0 (PMC10832242; doi:10.1186/s40104-023-00988-0)

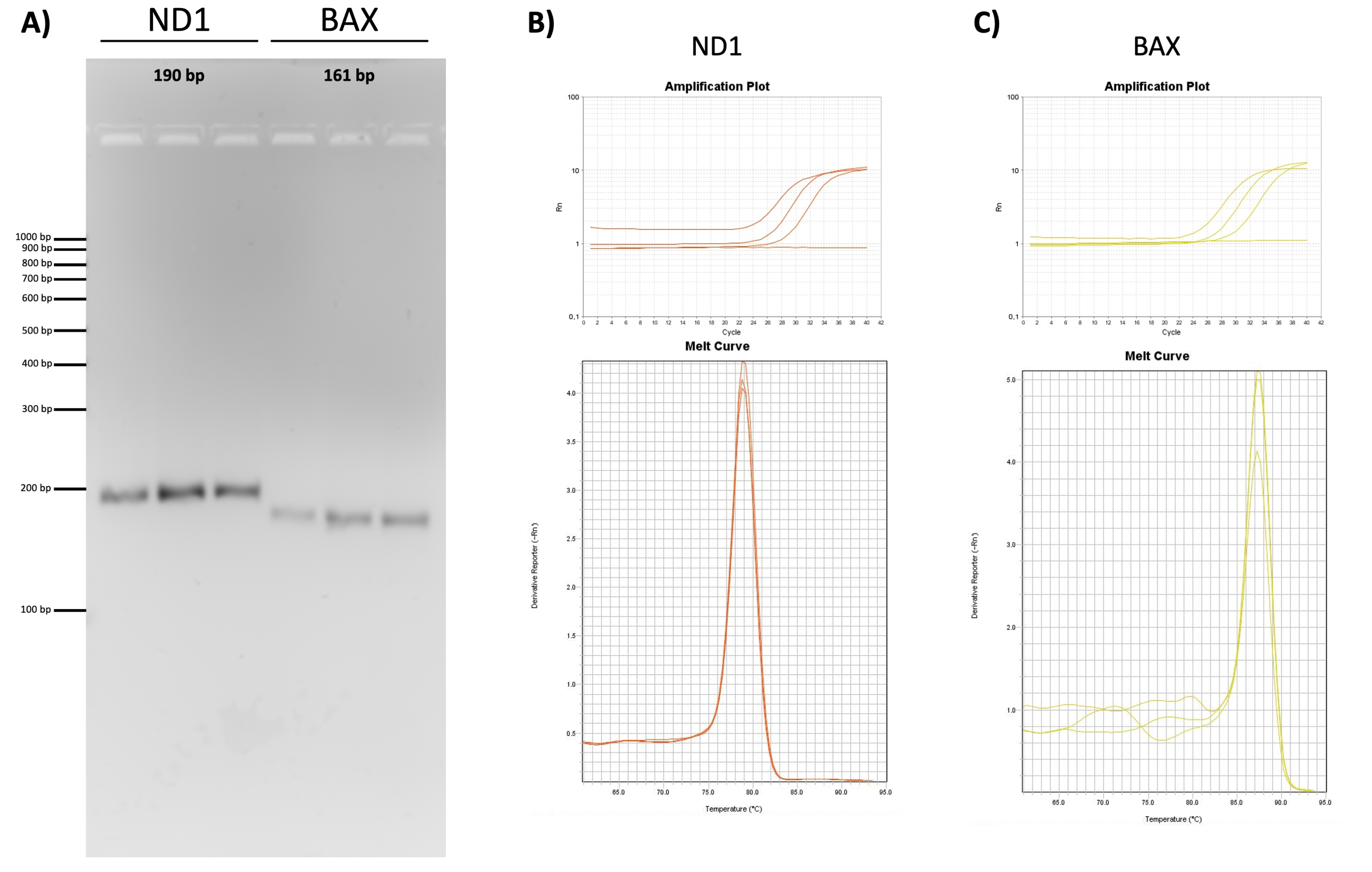

Supplement: Supplementary file 1 — Additional file 1. Melt curve analysis and 2% agarose gel electrophoresis of the PCR product resulting from the amplification of the NADH dehydrogenase subunit 1 (ND1) and the BCL2 Associated X (BAX) genes. [file 40104_2023_988_MOESM1_ESM.png]
