# Supplementary material for: Sperm function, mitochondrial activity and in vivo fertility are associated to their mitochondrial DNA content in pigs
Source: J Anim Sci Biotechnol. 2024 Feb 1;15:10. doi: 10.1186/s40104-023-00988-0 (PMC10832242; doi:10.1186/s40104-023-00988-0)

Additional file 2. Evaluation of the impact of density-gradient centrifugation (DGC) of semen on the mitochondrial DNA content (mtDNAc) in pigs. Fifteen pig seminal doses were pooled in groups of three (five biological replicates) and split into two subgroups: (i) control and (ii) DGC. DGC-selected samples were subjected to the procedure indicated by the manufacturer (BoviPure™; Nidacon, Mölndal, Switzerland). In parallel, the control underwent centrifugation under the same conditions as DGC-selected samples, but without the presence of BoviPure™. Subsequently, samples were resuspended in PBS. The percentage of non-cellular particles (debris), diploid cells, and haploid cells present in the control and DGC-selected samples were measured after incubation with Hoechst 34580 (H-34580; 6 μg H-34580 for 10^6 sperm at room temperature for 30 min) by flow cytometry (CytoFLEX cytometer; Beckman Coulter, Fullerton, CA, USA). H-34580 was excited with a 405 nm laser and the fluorescence was acquired with the PB450 channel (450/45). Finally, the mtDNAc of the two groups was quantified by qPCR of nuclear- (BCL2 Associated X; *BAX*) and mitochondrial- (NADH dehydrogenase subunit 1; *ND1*) encoded genes to determine the mtDNA content of each group. (**A**) Flow cytometry scatter plot of a representative sample of the control (A1) and DGC-selected samples (A2). The scatter plot provides information about the size (FSC) and shape (SSC), whereas the histogram shows the proportions of diploid cells, haploid cells, and debris particles based on H-34580 fluorescence intensity. (**B**) Mean and standard error of the mean (SEM) of the proportion of non-cellular particles (debris) (B1) , diploid cells (B2), and relative mtDNAc (B3) in control and DGC-selected samples. A paired t-test was performed to compare the two groups. (*) Indicates significant differences between groups ($P < 0.05$). The results suggest that pig seminal doses have a negligible proportion of non-spermatic cells, despite the presence of a significant amount of debris. The content of mtDNA in cells,

however, is statistically similar between control and DGC-selected samples, indicating that most mtDNA come from sperm cells.

A1

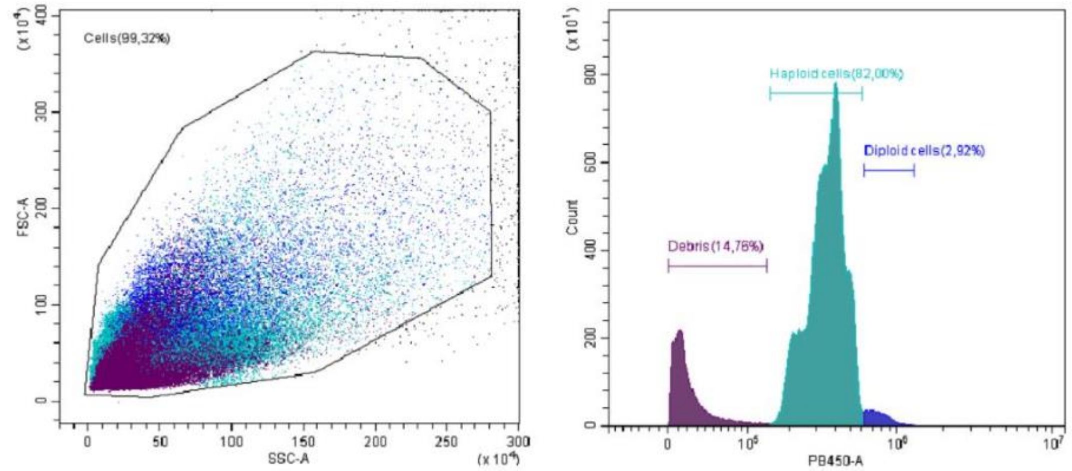

A2

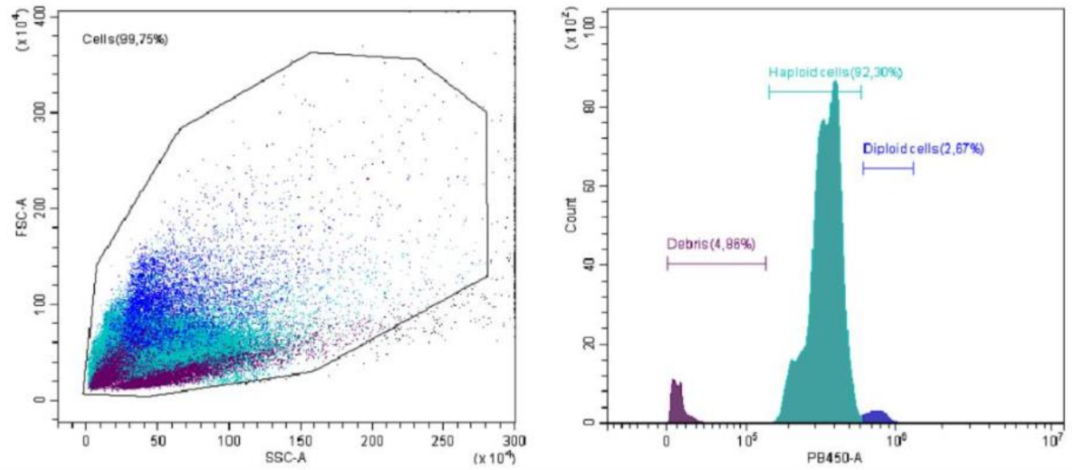

B1 **B2** **B3**

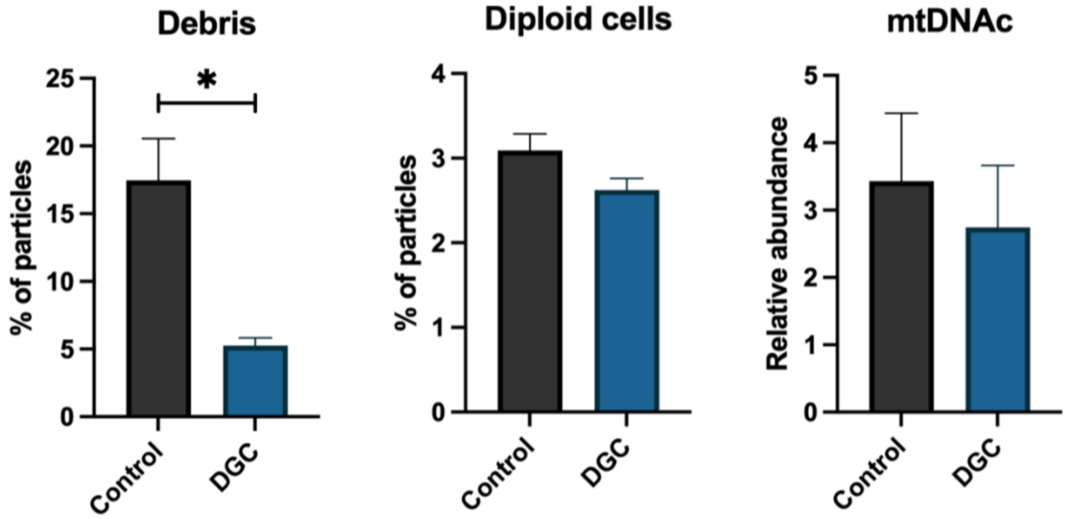

Supplement: Supplementary file 2 — Additional file 2. Evaluation of the impact of density-gradient centrifugation (DGC) of semen on the mitochondrial DNA content (mtDNAc) in pigs. [file 40104_2023_988_MOESM2_ESM.pdf]
